# A real-world study on clinical predictors of relapse after hospitalized detoxification in a Chinese cohort with alcohol dependence

**DOI:** 10.7717/peerj.7547

**Published:** 2019-08-28

**Authors:** Yu-Jie Tao, Li Hu, Ying He, Bing-Rong Cao, Juan Chen, Ying-Hua Ye, Ting Chen, Xia Yang, Jia-Jun Xu, Jing Li, Ya-Jing Meng, Tao Li, Wan-Jun Guo

**Affiliations:** Mental Health Center and Psychiatric Laboratory, West China Hospital, Sichuan University, Cheng Du, Si Chuan, People’s Republic of China

**Keywords:** Alcohol dependence, Relapse, Real-world study, Psychotherapy, Pharmaceutical treatment

## Abstract

**Background:**

The relapse rate of alcohol dependence (AD) after detoxification is high, but few studies have investigated the clinical predictors of relapse after hospitalized detoxification in real-world clinical practice, especially among Chinese patients.

**Methods:**

This longitudinal cohort study followed up 122 AD patients who were discharged from January 1, 2016 to January 30, 2018 from their most recent hospitalization for detoxification. These patients were interviewed by telephone from May 20, 2017, to June 30, 2018, at least 6 months after discharge. During the interview, the relapse were assessed by using a revised Chinese version of the Alcohol Use Disorder Identification Test. Candidate predictors, such as therapeutic modalities during hospitalization and at discharge, medical history data related to alcohol use, and demographic information, were obtained from the medical records in the hospital information system.

**Results:**

During the 6–24 months (with a median of 9 months) follow-up period, the relapse rate was 53.3%. Individuals with a college education level and those who had not been treated with the brief comprehensive cognitive-motivational-behavioural intervention (CCMBI) were more likely than their counterparts to relapse after hospitalized detoxification, and their adjusted HRs (95% CIs) were 1.85 (1.09, 3.16) and 2.00 (1.16, 3.46), respectively. The CCMBI use predicted a reduction in the relapse rate by approximately one-fifth.

**Conclusion:**

Undergoing the CCMBI during detoxification hospitalization and having less than a college-level education could predict a reduced risk of AD relapse. These findings provide useful information both for further clinical research and for real-world practice.

## Introduction

Excessive alcohol consumption not only contributes to functional impairment and decreased well-being of individuals but also results in a large public health burden worldwide. At the global level in 2016, alcohol consumption accounted for 5.3% (approximately 3 million) of all deaths worldwide and 5.1% of disability-adjusted life years (DALYs) lost (World Health Organization, 2018). One of the key reasons is that excessive drinking often leads to alcohol dependence (AD), which, as a chronic recurrent encephalopathy, has a disease course marked by repeated relapse. A recent systematic review gave the estimated current and lifetime prevalence of AD in China as 2.2% and 3.7%, respectively (Cheng et al., 2015). Follow-up studies have shown that the relapse rates of AD during the first 6 months after inpatient or outpatient treatment ranged from 42.9% to 60% in Western patient groups, and the rate of lapse (resumption of alcohol use) in the first 12 months after hospitalized detoxification was 100% in a group of Chinese AD patients (Addolorato et al., 2007; Moos & Moos, 2006; Weisner, Matzger & Kaskutas, 2003; Wojnar et al., 2009; Gao et al., 2018).

Many effective treatments for AD, both pharmaceutical and psychological, have been available in real-world addiction treatment settings. Pharmaceutical maintenance medications, such as disulfiram, acamprosate, naltrexone, nalmefene and topiramate, have been proven to have a mild-to-moderate effect in reducing relapse (Goh & Morgan, 2017; Li et al., 2017). Compared to pharmaceutical treatments, the acceptability of psychotherapy is better due to medication side effects concerns. There has been supportive evidence for the effectiveness of several psychotherapy modalities, including motivational interviewing (MI), cue exposure treatment, various cognitive-behavioral treatments, and brief interventions as effective psycho-social modalities in the treatment of alcohol problems (Martin & Rehm, 2012; Mellentin et al., 2016). There is also increasing evidence on the better relapse-prevention effect of a combination of pharmacotherapy and psychosocial therapy than pharmaceutical mono-therapy (Assanangkornchai & Srisurapanont, 2007; Connor, Haber & Hall, 2016). In China, some researchers also investigated the efficacy of electro-acupuncture aversion therapy for AD and found that this treatment effectively reduces the lapse rate (Jin, Li & Yang, 2006; Zhang, Zhang & Fan, 2014).

However, the real-world clinical application of the above-mentioned effective treatments for AD is still limited. First, the aforementioned treatment modalities were derived from randomized controlled trials (RCTs) with questionable external validity, because their subjects were highly selective and conditions were highly controlled, rather different from real-world clinical practice (Kennedy-Martin et al., 2015; Rothwell, 2005). Second, although some of the aforementioned pharmaceutical treatments have been recommended to prevent alcoholism relapse by the Chinese *Clinical Guideline of Diagnosis and Treatment for Alcohol Use Related Disorders* (Li et al., 2017), it still remains unclear whether they are applicable to Chinese AD patients. Specifically, most of the supporting evidence for these preventive medications has come from studies among Western patient groups and most of these medications are not readily available in China (i.e., disulfiram, acamprosate, and nalmefene). In addition, in China, many psychiatrists might use an integrated package of multiple evidence-based psychotherapy modalities to treat AD, but the literature contains little research on the studies of such comprehensive psychotherapeutic packages.

To fulfill these needs, the present study was set out to investigate alcohol use disorder relapse after hospitalized detoxification and its association with treatment modalities during hospitalized detoxification and at discharge in a Chinese patient group with AD.

## Methods

This real-world longitudinal cohort study was approved by the ethics committee of West China Hospital of Sichuan University in 2016 (No. 22), and verbal informed consent was obtained from each participant via telephone.

### Participants

This study followed up 122 alcohol-dependent patients who were discharged from January 1, 2016, to January 30, 2018, from their most recent hospitalization for detoxification in the Psychological Comprehensive Ward of the Mental Health Center, West China Hospital of Sichuan University, China. Each patient had a primary clinical diagnosis of a behavioural or mental disorder due to use of alcohol (coded F10.2-F10.5 in ICD-10) and met the diagnostic criteria for AD (coded F10.2 in ICD-10). Patients with intellectual disability, head trauma, any illicit substance abuse or dependence, or severe physical disease were excluded.

### Measurements

A case report form edited by the research group was used to collect the baseline data, including demographic information, alcohol resumption characteristics, and therapeutic modalities during hospitalization and at discharge.

### Baseline measurements

Information about demographic information (including age and education), the hospital length of stay (LOS) days and data related to alcohol use (including age of first drink, years of alcohol use, and a past history of hospitalization for alcohol detoxification) was collected from inpatient medical records.

This study was concerned especially with whether brain atrophy was associated with relapse, as there is sufficient evidence to indicate that brain atrophy is the most specific and common brain pathology found by clinical neuroimaging scans (Dupuy & Chanraud, 2016). Given that brain structure pathologies are common among alcoholic patients seeking hospitalized detoxification (Bjork, Grant & Hommer, 2003), all 122 participants in the present study had undergone structural neuroimaging using clinical magnetic resonance imaging (MRI) or computed tomography (CT). Thus the presence of brain atrophy was based on the result of MRI or CT at admission of each patient. Smoking status was determined by self-reporting. Participants who had never used nicotine or quitted for more than three months were considered non-smokers; the remaining patients were considered smokers.

During hospitalized detoxification, all participants received pharmacological therapies for symptomatic and supportive treatment. Among these pharmacological therapies, this study was interested in the use of psychotropic medications including benzodiazepines, antidepressants, antipsychotics, and antiepileptics, which were taken by a substantial part of the participants, not only during hospitalization but also after discharge. Previous studies have indicated that many comorbid mental/brain conditions (disorders or symptoms) such as depression, anxiety, psychoses, insomnia, and epilepsy play important roles in the development of alcoholism as either risks or outcomes, and pharmaceutical intervention for these conditions might be effective for alcoholism relapse prevention (Agabio, Trogu & Pani, 2018; Klimkiewicz et al., 2015; Li et al., 2017). Among the aforementioned medications identified by RCTs as “effective” medications for relapse prevention, only topiramate was used by a substantial portion of the participants because it was the only one readily available in the hospital. Thus, topiramate was the only specific individual medication investigated in this study.

In addition to routine psychological supportive intervention and relevant health education for all alcoholic inpatients, the inpatient ward also provided a voluntary basis brief comprehensive cognitive-motivational-behavioural intervention (CCMBI) package service for AD patients. This study thus investigated whether the patients had received CCMBI service as a psychotherapeutic variable to predict AD relapse.

### Outcomes

During the follow-up interview, all participants were asked “Have you ever drunk alcohol again since the latest discharge from the detoxification hospitalization?” Subjects who replied “yes” were further interviewed for their time (months) of the first re-drink and alcohol consumption of the most severe month after discharge using a revised Chinese version of the Alcohol Use Disorder Identification Test (AUDIT). The original Chinese version of AUDIT consists of 10 questions (total score range from 0 to 40) to estimate the severity of alcohol consumption in the preceding year (Babor, Higgins-Biddle & Robaina, 2014), different language versions (including the Chinese version) have been validated worldwide (Li et al., 2003). The timeframe of original Chinese AUDIT was revised from “twelve-month” to “one-month” for this study. Accordingly, this study defined a total AUDIT score ≥8 as relapse of alcohol use disorder (Babor, Higgins-Biddle & Robaina, 2014; Li et al., 2003). Time to re-drink was considered as the start point of relapse. The length (in months) of the interval between the last discharge and the follow-up interview (IDF) was also collected.

### Follow-up interview procedure

The follow-up interview for each participant was administered by an experienced psychiatric nurse and a psychiatry resident, both of whom were trained to ensure that the interview was standardized. They interviewed all 122 participants over telephone from May 20, 2017, to June 30, 2018, that is, at least 6 months after their discharge. All participants were given general health education to encourage them to remain abstinence or come to further clinical assessment for their relapse at the follow-up telephone interview.

### Statistical analysis

The rates and means (their 95% confidence intervals (95% CIs)) of baseline variables and outcomes were estimated using descriptive statistics. A univariate Cox proportional hazards regression model was implemented to assess the association between covariates and the probability of events (relapse) among individuals with AD. To determine the independent relapse predictors of AD, we performed multivariable Cox regression using probable predictors defined based on a two-tailed alpha level of 0.15 on univariate analysis as independent variables, as in a previous study (Boschloo et al., 2012). All statistical analyses were performed using the Statistical Package for the Social Sciences (SPSS 22.0 for Windows), and final results were evaluated based on a two-tailed alpha level of 0.05.

## Results

### Descriptive demographic and clinical features of participants

All patients were males. The age of the sample ranged from 25 to 73 years and was 44.6 (95% CI [42.8, 46.4]) years on average. Accordingly, the quartile groups aged 25–36 years, 37–44 years, 45–53 years, and 54–73 years included 30 (24.6%), 34 (27.9%), 36 (29.5%), and 22 (18.0%) participants, respectively. Their median duration of past alcohol use was 20 years. Their average LOS was 15.4 (95% CI [14.2, 16.5]) days, and their lengths of IDF ranged from 6 to 24 months with a median number of 9 months. The descriptive statistics of other clinical features were listed in Table 1.

### Rates of relapse

According to the follow-up interview, 53.3% (95% CI [44.3%, 62.3%]) of the participants were relapsed. The time from discharge to relapse ranged from 1 to 21 months, with a median of 2 months. The rates of relapse of each patient group by each categorical and dichotomous variable are listed in Table 1.

### Identification of predictors of relapse

Among the variables in univariate Cox regression models to predict relapse (including LOS, the length of IDF and other variables listed in Table 1), the experience of repeated hospitalization for alcohol detoxification (*p* = 0.048), having been treated with the CCMBI (*p* = 0.042), education level (*p* = 0.067) and being discharged with antipsychotics (*p* = 0.130) were identified as probable predictors.

**Figure 1:**
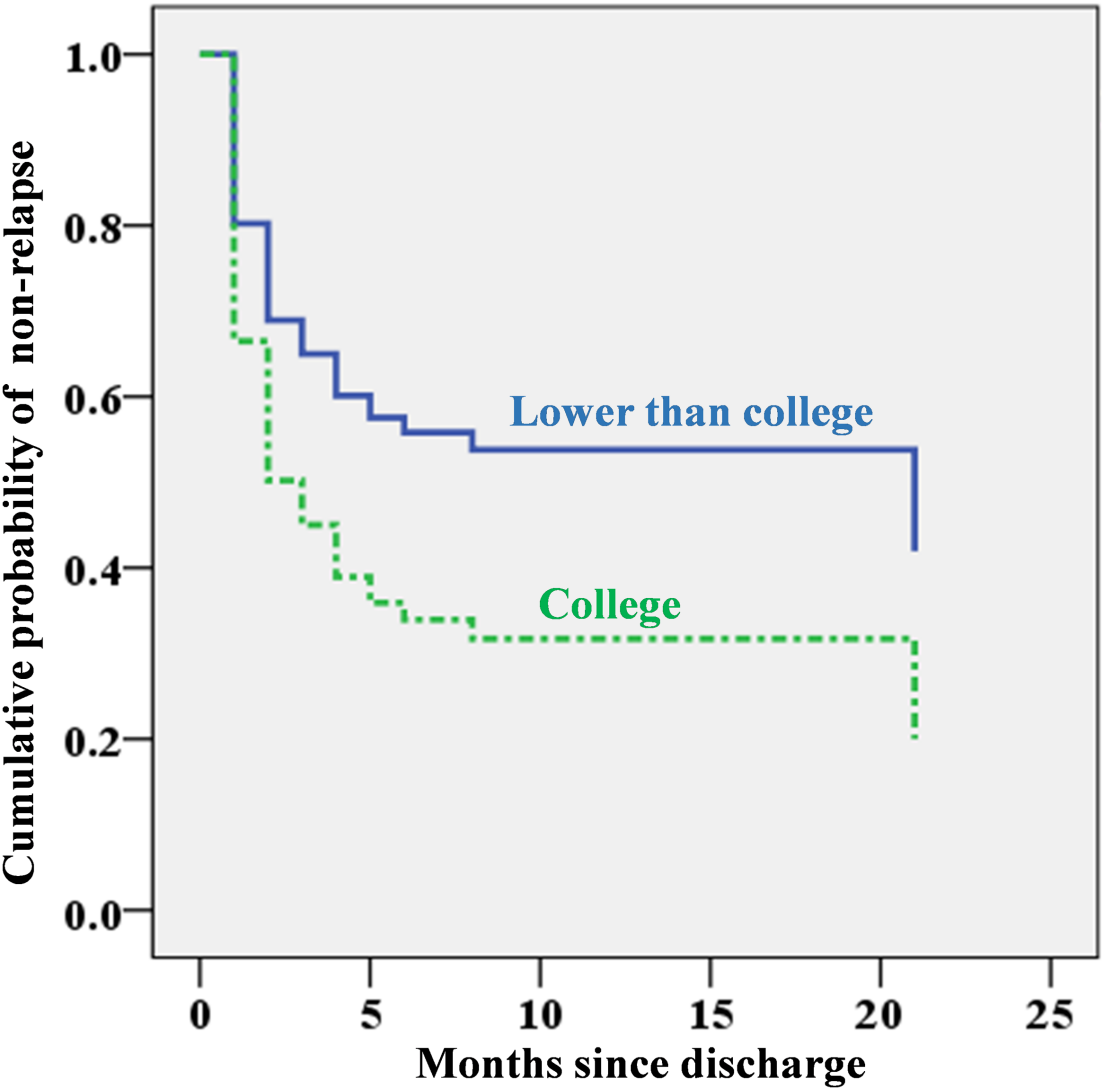
Cumulative probability of non-relapse since discharge by education level based on Cox regression analysis (using whether the patient had previously been hospitalized for alcohol detoxification, use of the CCMBI, and discharge with antipsychotics as covariates).

**Figure 2:**
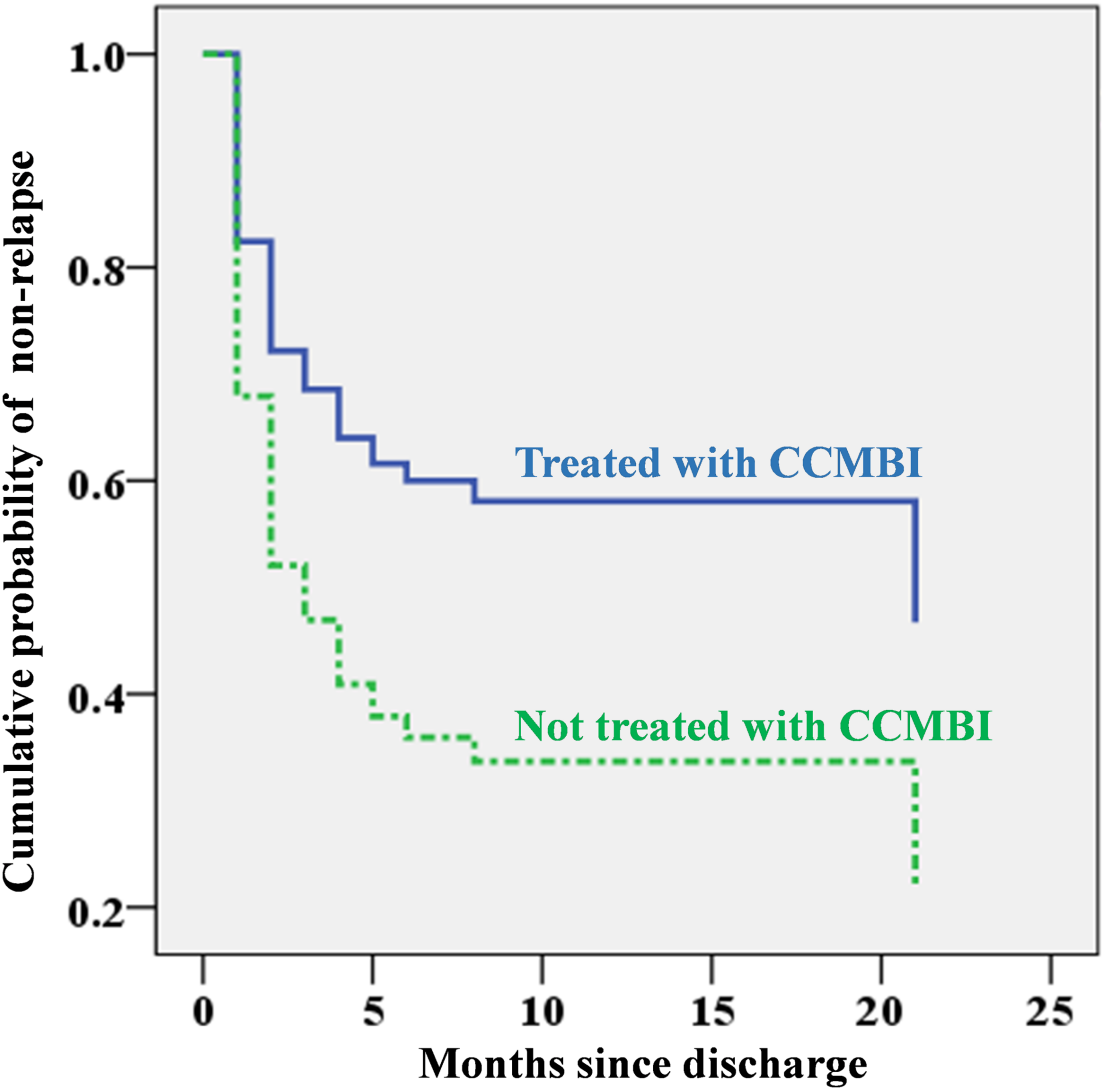
Cumulative probability of non-relapse since discharge by whether the combined cognitive-motivational-behavioural intervention (CCMBI) was applied during hospitalization for alcohol detoxification based on Cox regression analysis (using education category, whether the patient had previously been hospitalized for alcohol detoxification, and discharge with antipsychotics as covariates).

The multivariable Cox regression model to further explore the prediction property of these four probable predictors identified education level and CCMBI use as independent predictors. Individuals with a college education level and those who had not been treated with the CCMBI were more likely than their counterparts to relapse after hospitalized detoxification, and their adjusted HRs (95% CIs) were 1.85 (1.09, 3.16) and 2.00 (1.16, 3.46), respectively. The CCMBI use was associated with a reduction in relapse by approximately one-fifth in this study. The independent predictive effects of education level and CCMBI for relapse are depicted in Figs. 1 and 2.

**Table 1:** The relapse^a^ rate (%) after hospitalized alcohol dependence detoxification and its associations with demographic and clinical features.

	**Sample ratio (%)**	**Relapse rate (95% CI)**^b^	**Univariable model**		**Multivariable model**
			**X**^2^^c^	**HR**^d^**(95% CI)**^b^	**aHR**^e^**(95% CI)**^b^
Total (*n* = 122)	100	53.28 (44.30, 62.26)		–	–
Age group (years)	–		0.63	–	–
25–36	24.59	56.67 (37.85, 75.49)		1	–
37–44	27.87	50.00 (32.29, 67.71)		0.90 (0.46, 1.77)	–
45–53	29.51	58.33 (41.42, 75.25)		1.00 (0.53, 1.90)	–
54–73	18.03	45.45 (22.86, 68.05)		0.76 (0.35, 1.66)	–
Education level	–		3.42^**^		
Lower than college	62.30	47.37 (35.88, 58.85)		1	1
College	37.70	63.04 (48.55, 77.54)		1.59(0.97,2.60)^**^	1.85 (1.09, 3.16)^*^
Years of alcohol use	–		0.12		
Up to 20 years	50.00	50.82 (37.91, 63.73)		1	–
More than 20 years	50.00	55.74 (42.91, 68.56)		1.09(0.67,1.78)	–
Smoker	–		0.00		
Yes	89.34	55.05 (45.56, 64.53)		1.03(0.47,2.25)	–
No	10.66	38.46 (7.86, 69.06)		1	–
First-time hospitalization for alcohol detoxification	–		4.00^*^		
Yes	57.38	45.71 (33.75, 57.68)		1	1
No	42.63	63.46 (49.94, 77.00)		1.64 (1.01, 2.67)^*^	1.41 (0.85, 2.33)
Brain atrophy	–		0.78		
Yes	30.33	45.95 (29.10, 62.79)		0.78 (0.45, 1.36)	–
No	69.67	56.47 (45.71, 67.23)		1	–
Treated with CCMBI^f^	–		4.24^*^		
Yes	47.54	43.10 (29.97, 56.24)		1	1
No	52.46	62.50 (50.31, 74.69)		1.69 (1.02, 2.78)^*^	2.00 (1.16, 3.46)^*^
Discharge with BDZ^g^	–		0.74		
Yes	45.90	57.14(43.77,70.52)		1	–
No	54.10	50.00 (37.61, 62.39)		0.81 (0.50, 1.31)	–
Discharge with antipsychotics	–		2.32^**^		
Yes	66.39	48.15 (37.03, 59.27)		1	1
No	33.61	63.41 (48.02, 78.81)		1.47 (0.89, 2.41)^**^	1.43 (0.85, 2.40)
Discharge with antidepressants	–		0.26		
Yes	26.23	50.00 (31.68, 68.32)		1	–
No	73.77	54.44 (43.96, 64.93)		1.16 (0.66, 2.04)	–
Discharge with topiramate	–		0.09		
Yes	53.28	53.85 (41.40, 66.29)		1	–
No	46.72	52.63 (39.27, 66.00)		0.93 (0.57, 1.51)	–
Discharge with antiepileptics	–		0.21		
Yes	83.61	51.96 (42.10, 61.82)		1	–
No	16.39	60.00 (36.48, 83.52)		1.16 (0.62, 2.17)	–

**Notes.**

a Relapse: defined using the revised Chinese version of Alcohol Use Disorder Identification Test (AUDIT), assessing the alcohol consumption in the highest-severity month; the cutoff is ≥8.

b 95% CI, 95% confidence interval.

c Based on Cox proportional hazards regression analysis.

d HR, Hazard ratio based on univariate Cox proportional hazards regression analysis.

e aHR, Adjusted hazard ratio based on multivariable Cox proportional hazards regression analysis; factors with *P* < 0.15 in the univariate analyses were entered into the statistical model.

f Comprehensive cognitive-motivational-behavioural intervention (CCMBI): CCMBI was developed primarily based on the WHO manual for brief intervention for hazardous and harmful drinking, and added components of motivational interviewing, cue exposure, and aversion therapy.

g BDZ: Benzodiazepines.

* *P* < 0.05.

** *P* < 0.15.

## Discussion

To our knowledge, this study is the first of its kind to explore real-world therapeutic predictors of alcoholism relapse, including both pharmaceutical and psychological predictors, during the months following hospitalized detoxification (at least six months of follow-up) in more than 100 patients. By doing so, this study found that the rate of alcohol use disorder relapse (53.3%) after detoxification in China was comparable to the international rates. The relapse rate found in this study was in the range of relapse rates of treated AD (from 42.9% to 60%) reported in different studies (Addolorato et al., 2007; Moos & Moos, 2006; Weisner, Matzger & Kaskutas, 2003; Wojnar et al., 2009).

The most exciting finding of this study was that ongoing CCMBI during detoxification hospitalization was a statistically significant predictor for less relapse of alcoholism. The CCMBI was associated with a reduction in relapse by approximately one-fifth and resulted in a HR of 2.00 in this study. A partial explanation for this effect size may be that the treatment itself was an integration of multiple components of effective psychotherapies, including components of the WHO manual for brief intervention for hazardous and harmful drinking, MI, cue exposure, and aversion therapy (Li et al., 2017; Miller & Rollnick, 2012; McQueen, Ballinger & Howe, 2017; Penberthy et al., 2011). However, these results should be interpreted with caution because patients who consent to receive the CCMBI in real-world clinical practice may have stronger motivation to accept and comply with treatment—and may consequently have a better prognosis—than their counterparts (Bauer, Strik & Moggi, 2014). Additional strictly designed RCT studies are needed to test the real therapeutic efficacy of the CCMBI. Nonetheless, integrating the findings of this study with the previously documented evidence that psychotherapy improved the prognosis of AD (Martin & Rehm, 2012; Mellentin et al., 2016; Jin, Li & Yang, 2006; Zhang, Zhang & Fan, 2014), the CCMBI is worthy of recommendation for use in routine real-world clinical practice.

A surprising finding of this study is that college-level education, compared to a lower education level, was a predictor for relapse. Previous studies usually reported that a lower education level was associated with a higher relapse rate (Bottlender & Soyka, 2005; Moos & Moos, 2006), supporting a popular interpretation wherein individuals with a lower education level have poorer awareness or knowledge of alcohol-related health problems (Moos & Moos, 2006). However, Boschloo et al. (2012) have found that a high education level was a significant risk factor for the persistence of AD. The reasons for a higher education level being associated with relapse of AD or persistence of AD are complex but could be at least partly explained as follows: First, assuming that individuals with a higher education level may have already had greater awareness or knowledge of alcohol-related health problems, they might thus receive less benefit from health education, which is considered as an important component of therapeutic intervention for AD (WHO, 2001). Second, the aetiology of AD in patients with a higher education level might be attributable in larger part to factors other than poor health awareness or knowledge, such as stress, family history of AD, and genes that could decrease the response to treatment or prevention. Last but not least, the social and cultural circumstances in China made alcoholic beverages a popular element of social occasions, especially for highly educated groups, and patients with higher education may have more “social burdens and pressure” to drink or be exposed to more cues related to alcohol use (Zhao, 2006).

Unexpectedly, this study did not find any investigated pharmaceutical interventions that were significantly associated with relapse of AD, even in the case of topiramate, whose effectiveness in reducing AD has been repeatedly identified by published research (Blodgett et al., 2014; Goh & Morgan, 2017). However, the findings regarding these medications’ lack of preventive effectiveness against AD relapse need to be interpreted carefully due to the following relevant limitations of this real-world study: First, this study analysed medications prescribed at discharge but did not assess patients’ compliance with the prescriptions. Second, most investigated medications in this study were used to address or prevent comorbid mental disorders or symptoms, which themselves might influence the prognosis of AD (Schellekens et al., 2015; Suter, Strik & Moggi, 2011). This study, however, failed to control such confounding effects due to the lack of systemic, standardized, or structured measurements for comorbid disorders or symptoms in real-word clinical practice. From this point of view, more measurement-based clinical practice would improve the quality of real-world clinical research. In addition, the sample size of this study is insufficient to generate statistical significance for predictors with small effect size.

This study has several other limitations in addition to the ones mentioned above. As the participants were AD patients who had experienced hospitalized detoxification, this study may have been be biased towards severe cases, causing the probability of relapse to be overestimated (Cohen, 1984). Because patients were recruited from only one hospital, the study might lead to selection bias. Although measurements of AD relapse through telephone interviews have been validated and used in some previous studies (Glass et al., 2017; Moos & Moos, 2006), they could be further validated in this Chinese patient group with the assistance of laboratory test indicators, such as serum ethanol concentration and carbohydrate-deficient transferrin (CDT) (Bortolotti et al., 2018). As the participants were all males, the findings of this study should not be generalized to female patients, although a recent review indicated that the relapse rate of AD was similar between genders (Petit et al., 2017).

## Conclusion

The aforementioned limitations notwithstanding, this study found that the brief CCMBI during detoxification hospitalization and an education level lower than college predicted a reduced risk of AD relapse. These findings provide useful information both for further clinical research and for real-world practice.

##  Supplemental Information

10.7717/peerj.7547/supp-1 Supplemental Information 1 Alcohol dependence real-world research

## Additional Information and Declarations

### Data Availability

The following information was supplied regarding data availability: Data is available as Supplemental File.
